# Chasing ChEs-MAO B Multi-Targeting 4-Aminomethyl-7-Benzyloxy-2*H*-Chromen-2-ones

**DOI:** 10.3390/molecules24244507

**Published:** 2019-12-09

**Authors:** Mariagrazia Rullo, Marco Catto, Antonio Carrieri, Modesto de Candia, Cosimo Damiano Altomare, Leonardo Pisani

**Affiliations:** Department of Pharmacy—Drug Sciences, University of Bari “Aldo Moro”, via E. Orabona 4, 70125 Bari, Italy

**Keywords:** acetylcholinesterase inhibitors, butyrylcholinesterase inhibitors, monoamine oxidase B inhibitors, multi-target-directed ligands, coumarin derivatives, structure–activity relationships, docking simulations

## Abstract

A series of 4-aminomethyl-7-benzyloxy-2*H*-chromen-2-ones was investigated with the aim of identifying multiple inhibitors of cholinesterases (acetyl- and butyryl-, AChE and BChE) and monoamine oxidase B (MAO B) as potential anti-Alzheimer molecules. Starting from a previously reported potent MAO B inhibitor (**3**), we studied single-point modifications at the benzyloxy or at the basic moiety. The in vitro screening highlighted triple-acting compounds (**6**, **8**, **9**, **16**, **20**) showing nanomolar and selective MAO B inhibition along with IC_50_ against ChEs at the low micromolar level. Enzyme kinetics analysis toward AChE and docking simulations on the target enzymes were run in order to get insight into the mechanism of action and plausible binding modes.

## 1. Introduction

**Figure 1:**
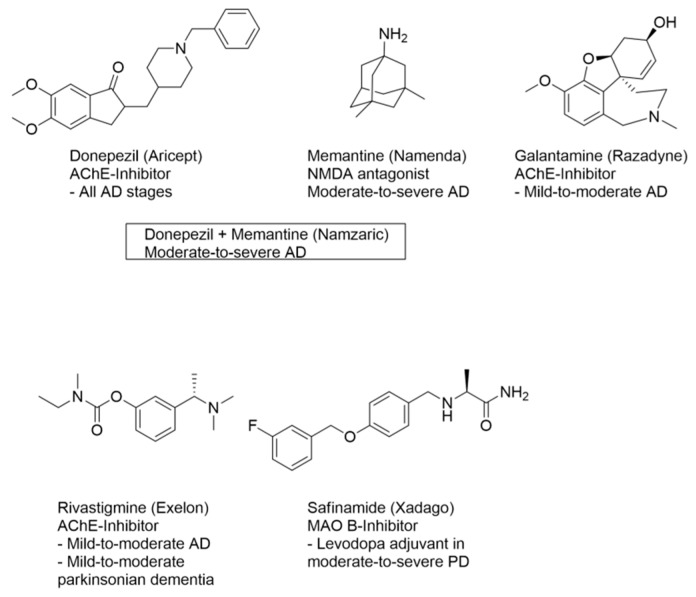
Drugs and combinations approved for the treatment of Alzheimer’s disease (AD).

Alzheimer’s disease (AD), the main cause of dementia, represents the sixth leading cause of death in US, killing more patients than breast and prostate cancer combined [1]. Nowadays, most AD cases are distributed to higher-income countries, but projections indicate that a prevalence increase will take place in both low- and middle-income countries in the next two decades [2]. Even if more than one century has passed from the seminal observation made by Alois Alzheimer, the great efforts devoted to the understanding of AD aetiology are still not comprehensive [3]. As a matter of fact, all the approved drugs exert palliative benefits by attenuating the cognitive symptoms (e.g., memory loss) in mild cases, but they lose efficacy in the late stages of the disease when the progressive loss of neurons results ultimately fatal [4]. Since the neuronal impairment is initially restricted to cholinergic areas, with the exception of memantine, three out of the four marketed drugs, as seen in Figure 1, aim at restoring basal neurotransmitter levels, namely acetylcholine (ACh), by means of inhibiting acetylcholinesterase (AChE) [5]. More recently, for mild-to-severe AD cases, the FDA approved the first drug combination that includes two active ingredients with different mechanisms of action, that is donepezil (AChE inhibitor) and memantine (glutamate-receptor blocker) [6]. This polypharmacological approach strengthens the idea that multifactorial neurodegenerative disorders (NDs), including AD, might be more efficiently handled with therapeutic protocols able to modulate two or more etiopathological mechanisms. In fact, NDs share the multifactorial nature of cardiovascular and tumor diseases, which are commonly treated with drug cocktails or combinations. The challenging goal of disclosing a single molecular tool endowed with multiple disease-related pharmacological activities represents the spirit of the so-called multi-target strategy [7].

In this study, we aimed at discovering novel multipotent anti-AD pharmacological tools through the identification of multimodal compounds capable of counteracting cholinergic depletion and oxidative stress conditions, by inhibiting ChEs and monoamine oxidase B (MAO B), as part of our long-standing project. Neuroinflammation, a typical condition of AD brains, is, among others, sustained by MAO B, whose upregulation in reactive astrocytes results in aberrant GABA levels [8] and reactive oxygen species (ROS) production through the catalytic deamination of the starting amine substrate into an aldehyde metabolite and hydrogen peroxide as the byproducts [9]. While searching for new anti-Parkinson agents, we previously reported compound **3** (alias NW-1772, compound **22b** in ref. [10]) that behaved as a highly selective (SI, IC_50_ MAO A/IC_50_ MAO B = 457) and in vivo potent rat MAO B inhibitor, with affinity in the nanomolar range (IC_50_ rat MAO B = 13 nM), rapid blood–brain barrier penetration, and poor CYP450s liability. The X-ray crystal structure of **3** in complex with hMAO B showed a binding mode in the aromatic cavity superimposed to that of safinamide (Figure 1) [11], recently marketed as an anti-Parkinson drug in several EU countries. The crystallographic pose within the enzymatic cleft showed the protonatable substituent placed close to FAD coenzyme in the catalytic site whereas the benzyloxy group is anchored to the entrance cavity.

**Figure 2:**
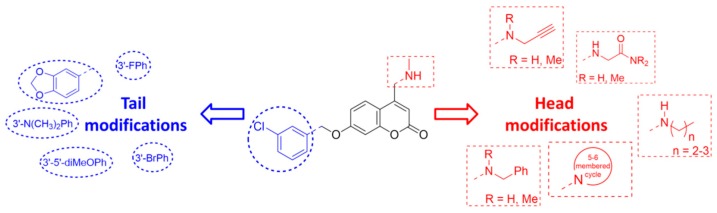
Structural modifications of starting hit NW-1772 (**3**).

As a follow-up of our research on small-molecule agents for treating AD [12], we have recently focused on single- [13] and multi-targeting coumarin derivatives [14]. The coumarin (2*H*-chromen-2-one) nucleus represents a nature-friendly, easy-to-handle scaffold that has been widely explored to hit several targets depending on the chemical decoration around the heterocyclic skeleton, including antimicrobial [15], anticoagulant [16], antiproliferative [17], antiangiogenic [18], and anti-HIV agents [19], among others. We found that compound **3** retained single-digit nanomolar inhibitory activity and great selectivity toward human MAO B (IC_50_ human MAO B = 3.9 nM, SI = 167) and it was endowed with moderate affinity toward AChE (IC_50_ = 6.2 µM) and lower inhibitory potency toward BChE (43% inhibition at 10 µM). Encouraged from in vitro data toward ChEs, herein, the approach to get novel multipotent molecules started from hit **3** by introducing head (substituent at position 4 of coumarin, coloured in red in Figure 2) or tail modifications (benzyloxy group, blue-coloured in Figure 2) at the 4-aminomethyl-7-benzyloxycoumarin core, rooted on X-ray complex analysis. In the first case, differently sized basic moieties were attached at C4 of the coumarin core while maintaining the 3′-chlorobenzyloxy group at C7. In the second subset, the unhindered C4-substituent (namely the methylaminomethyl group) was kept untouched and a number of different substituents were introduced around the phenyl ring belonging to the benzyloxy tail. To widen the structure–activity relationships, small groups (e.g., Me, Cl) were inserted at positions 6 and 8 of the parent skeleton of **3**. All compounds were screened toward human MAOs and ChEs (AChE from electric eel and BChE from equine serum). In this regard, MAO B selectivity was pursued to avoid safety issues related to the so-called “cheese-effect” whereas, on the other side, AChE/BChE selectivity was not deemed critical since increasing lines of evidence supported the involvement of BChE in chronic phases of AD thus claiming for BChE as a promising therapeutic target [20].

## 2. Results

### 2.1. Chemistry

**Scheme 1:**
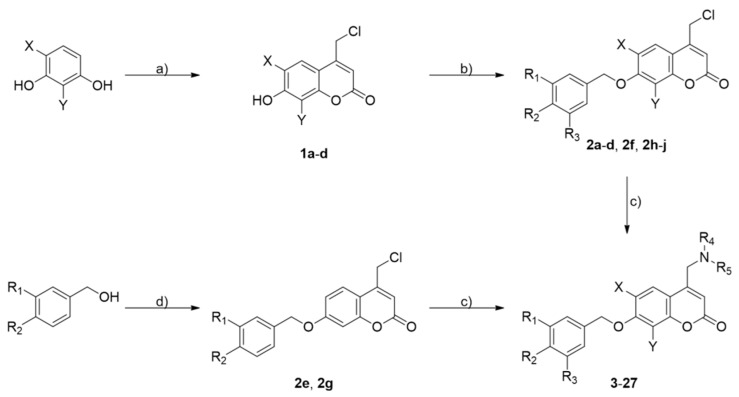
Reagents and conditions: a) ethyl 4-chloroacetoacetate, conc. H_2_SO_4_, 0 °C for 1–2 h (for **1a**, **1c**) or room temperature for 24 h (for **1b**, **1d**); b) suitable benzyl bromide, *N,N-*diisopropylethylamine (DIEA), ethanol, 1.5–5 h, Δ; c) appropriate amine, dry tetrahydrofuran (THF) or dimethylformamide (DMF), DIEA or K_2_CO_3_, 4–72 h, room temperature or 80 °C; d) 4-(chloromethyl)-7-hydroxy-2*H*-chromen-2-one (**1a**), diisopropyl azodicarboxylate (DIAD), PPh_3_, dry THF, 18 h, room temperature.

Scheme 1 illustrates the synthetic route to obtain the final coumarin derivatives **3**–**27**. Von Pechmann cyclization on substituted 1,3-dihydroxybenzenes afforded the appropriate 4-chloromethyl-7-hydroxy coumarin **1a**–**d**, which was alkylated with the suitable benzyl bromides before reacting with primary or secondary amines to yield desired final compounds **3**, **6**–**27**. A different route was followed to prepare coumarin **4**–**5** through intermediates **2e** and **2g**, requiring the alkylation of **1a** through a Mitsunobu etherification with commercially available piperonyl alcohol or **1e**, which was in turn obtained from borane-promoted reduction of 3-(dimethylamino)benzoic acid.

### 2.2. In Vitro Screening

To identify novel multipotent inhibitors, the in vitro screening program started from evaluating some congeners of **3** recruited from an in-house library of coumarin derivatives (**6**–**9**, **13**–**15**, **17**–**21**), previously tested as rat MAOs inhibitors [10,21]. Moreover, newly synthesized compounds (**4**–**5**, **10**–**12**, **16**, **22**–**27**) were screened toward the target enzymes (MAOs and ChEs) in order to derive structure–activity relationships focused on the multi-targeting features of 4-aminomethyl-7-benzyloxy-2*H*-chromen-2-ones. In vitro enzymatic screening toward ChEs was carried out according to the known Ellman’s protocol [22], by using eeAChE and esBChE as highly homologous and cheaper surrogates for human enzymes. The enzyme inhibition assays on human MAOs were performed by applying already reported methods [14]. The biological data for all derivatives are collected in Table 1.

## 3. Discussion

### 3.1. Structure–Activity Relationships

Structural modifications around the benzyloxy-tail region markedly affected MAO B affinity. Indeed, the presence of hydrogen-bonding acceptor (HBA) groups at position 3 (**4**) or 3,5 (**5**) reduced MAO B affinity and selectivity. These substitution patterns were scarcely tolerated from both ChEs as well. Interestingly, the presence of the piperonyl group (**6**) or *meta*-halogens (e.g., Cl in **3**, Br in **8**, and F in **9**) produced MAO B selective compounds almost equipotent to the unsubstituted benzyloxy-derivative **7**. This compound was the least active ChEs inhibitor (26% and 17% of inhibition at 10 µM toward AChE and BChE, respectively). Conversely, halogens at the *meta* position improved the inhibitory activity against both AChE and BChE, particularly Br and F.

When little modifications were applied around the heterocyclic backbone, the inhibition of ChEs was moderate-to-poor and MAO B/A isoform selectivity fell down drastically. A methyl group at position 6 or 8 produced an affinity drop toward MAO B compared to **3**. A more dramatic decrease was returned by Cl at position 6.

Then, the investigation was shifted to the modification of the basic protonatable head. As expected from crystallographic poses, the steric hindrance of the basic head played a detrimental role in the binding interactions with hMAO B. Confirming the prediction of a 3D-QSAR model previously developed on rat enzymes [23], bulky groups (benzyl in **13**–**14**, aliphatic 5–6 membered nitrogen-containing cycles in **15**–**17**) led to a significant reduction of MAO B inhibitory potency compared to **3**. Both molecular simplification of the basic head into primary amine **18** and the modification of the basic head into a tertiary amine, albeit unhindered, did not ameliorate the multi-targeting activity (compare **14** with **13**, **23** with **22**, **18**–**19** with **3**). In addition, tertiary amines (**14**, **19**, and **23**) displayed a lower B/A selectivity. The homologation of the alkyl chain on the basic nitrogen of **3** to linear *n*-propyl (**20**) and *n*-butyl groups (**21**) progressively reduced MAO B inhibitory potencies (**3** < **20** < **21**), too. The introduction of a terminal propargyl group led to a potent and selective MAO B inhibitor (**22**) endowed with a three-fold improved AChE affinity compared to **3** (IC_50_ values equal to 2.1 and 6.2 µM, respectively) and a slightly lower BChE inhibition. Finally, glycine-inspired terminal chains were introduced (**24**–**26**). These coumarins were potent MAO B inhibitors, with compound **25** being a more potent MAO B inhibitor than **3** (IC_50_ = 1.1 nM). Within this subset, it is worth noting that derivative **25** represented the most selective MAO B inhibitor of the whole series and **24** showed no selectivity at all, providing nanomolar inhibition of both MAO isoforms (IC_50_ = 77 and 61 nM toward MAO A and B, respectively). The increasing hindrance on the terminal amide and/or the reduced HB networking did not clearly correlate with dual AChE/MAO B affinity. Moreover, these polar chains were not tolerated by BChE.

In order to probe potential additive effects, we selected and combined one head and tail modification among the previous ones by joining a 3,5-dimethoxybenzyl group (from **5**) to a Gly-NH_2_ fragment (from **24**) in the same molecule. Interestingly, the resulting derivative **27** showed a higher MAO B affinity and selectivity than **5** and **24**, whereas ChEs inhibition was from moderate (AChE) to low (BChE).

**Figure 3:**
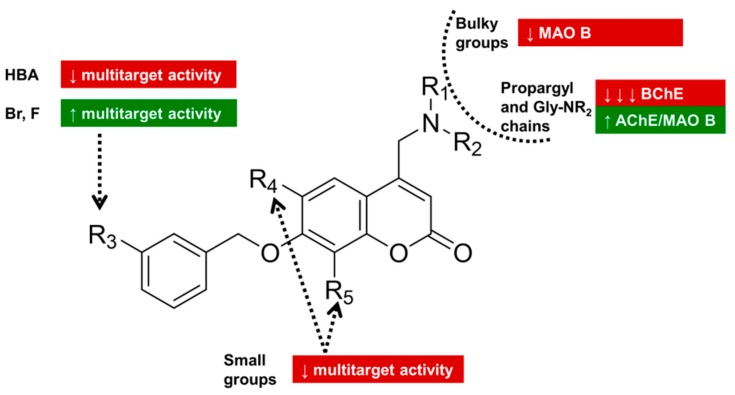
Pictorial representation of structure–activity relationships.

Concerning trends in structure–activity relationships, as seen in Figure 3, MAO B affinity is much more sensitive than AChE to modifications at the protonatable moiety, where bulkier groups and unhindered tertiary amines led to less active MAO B inhibitors. Halogens (particularly F and Br) in the *meta* position of the benzyloxy tail represent the most effective substituents for good triple targeting activity and high B/A selectivity.

Upon looking for MTDLs, an appropriately balanced bioactivity ratio is a challenging, yet desirable, goal [24]. Most of the inhibitors described herein missed this outcome, since their inhibitory readouts were biased against MAO B, and their IC_50_ values differ by more than one order of magnitude, showing nanomolar MAO B affinity along with moderate ChEs inhibition in the micromolar range (**6**, **8**, **9**, **20**). Instead, compound **16** was endowed with a potent and well-balanced bioactivity profile (submicromolar inhibition of MAO B, low micromolar inhibition of AChE and BChE), showing AChE/MAO B and BChE/MAO B IC_50_ ratio equal to 11 and 9, respectively.

As reported in Table 2, these multimodal hit compounds were endowed with encouraging drug-like features, showed no violation of Lipinsky’s RO5, and they were predicted to be CNS permeant.

### 3.2. AChE Kinetics

**Figure 4:**
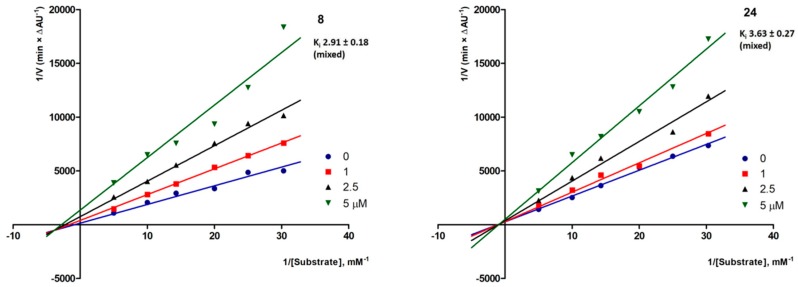
Electric eel AChE enzyme kinetics for compounds **8** (left) and **24** (right).

Enzyme kinetics were used as tool for investigating the inhibition mechanism toward AChE. Compounds **8**, bearing a *meta*-bromobenzyloxy substituent at coumarin C7, and **24**, branched with a glycinamide chain as the variation at position 4, were chosen as prototypes bearing different head-or-tail modification schemes. Both compounds showed among the highest AChE inhibitory potencies (IC_50_ in the low micromolar range). Similarly, with donepezil [25], Lineweaver–Burk plots in Figure 4 indicated a mixed-type mechanism of inhibition toward eeAChE, which suggested the possibility of occupying the peripheral anionic subsite (PAS) region, at least in part. Moreover, compound **8** showed a K*_i_* equal to 2.91 µM and derivative **24** returned a K*_i_* = 3.63 µM.

The chance of occupying the PAS of AChE acquires relevance in relation to AD, where the chaperone activity of this region enhancing the fibrillization of neurotoxic amyloid monomers has been well-documented [26].

### 3.3. Docking Studies

In order to get clues regarding a plausible binding mode within target enzymes, docking simulations were run for compounds **8** and **24**. As it could have been expected from the high similarity with compound **3**, the binding modes within hMAO B enzymatic cleft were close to those of the cocrystallized ligand (PDB entry 2V61, data not shown) [11].

**Figure 5:**
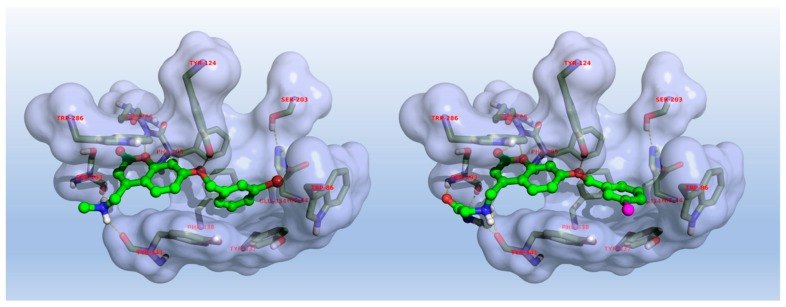
Predicted binding mode of compounds **8** (left) and **24** (right) within hAChE (PDB entry 6O4W). For a data summary, see Table S1.

Aiming at studying binding interactions with AChE, the hAChE/donepezil X-ray complex (PDB entry 6O4W) [27] was enrolled to this end, given that hAChE and eeAChE, used for in vitro screening, share 88% identity and 93% overall sequence homology [28]. As shown in Figure 5, in both instances, the heterocyclic cores were accommodated at the PAS in a way that most likely resembled the pose of the native ligand (e.g., donepezil), as seen in Figure S1, even if the coumarin ring twisting did not accomplish a proper geometry matching Trp286 for an optimal face-to-face π–π interaction. Nonetheless, the whole molecular architecture was further stabilized by a network of polar contacts (hydrogen and π-cation bonds) anchoring the basic heads to residues lining the entrance of the active-site gorge (e.g., Ser293, Trp286). Additional hydrophobic interactions were captured by **8** and **24** with the side chains of Phe338, Tyr337, and Tyr341. Interestingly, the same outcomes were obtained when applying a different X-ray complex (PDB entry 6EY6) [29], where galantamine is totally embedded within the catalytic region and a different rotamer was described for Tyr337 (Phe330 in eeAChE), pointing toward the mid-gorge, as seen in Figure S2. In these runs, compounds **8** and **24** were unable to occupy either the catalytic region, or to fit CAS (data not shown), showing similar binding epitopes pointing to the outer mid-gorge region and Trp286 at the PAS.

**Figure 6:**
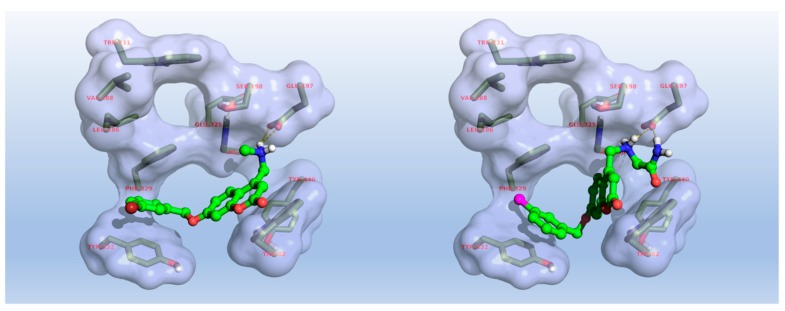
Predicted binding mode of compounds **8** (left) and **24** (right) within human BChE (PDB entry 6F7Q).

Regardless of its large structural homology (about 70%) with AChE, BChE features a larger cavity (almost 200 Å^3^), making it accessible to bulkier binders. Docking studies involving hBChE (PDB entry 6F7Q) retrieved plausible binding poses for coumarin **8**, as seen in Figure 6, suggesting the ability of this derivative to suitably interact with aromatic residues at CAS (Trp82) and PAS (Tyr332) of BChE. On the other side, compound **24** adopted an unsuitable coumarin-ring twist for interacting with Trp82, thus showing lower anti-BChE activity, as can be seen for compounds **24**–**27** bearing differently substituted Gly-NH_2_ side chains.

## 4. Materials and Methods

### 4.1. Enzyme Inhibition Assays

All enzymes and reagents were purchased from Sigma Aldrich, Milan, Italy. Inhibition of human recombinant MAO A and B was assayed by monitoring the fluorescence of 4-hydroxyquinoline produced from the oxidation of substrate kynuramine, following a previously published method [14]. Acetylcholinesterase from electric eel and butyrylcholinesterase from equine serum were used in the Ellman’s spectrophotometric method as already described [13]. In all cases, a 96-well plate procedure was used with an Infinite M1000 Pro plate reader (Tecan, Cernusco sul Naviglio, Italy). Inhibition values were determined as IC_50_ values by statistical analysis using Prism (GraphPad Prism version 5.00 for Windows, GraphPad Software, San Diego, CA, USA). For less active compounds, percent inhibition was measured at 10 μM. Inhibition kinetics of eeAChE were determined as described [13], using four concentrations of inhibitor (0 to 5 μM) and six concentrations of substrate acetylthiocholine (33 to 200 μM).

### 4.2. Molecular Modeling

Compounds **8** and **24**, with standard values of bond lengths and valence angles, were built within the Maestro software package [30] as protonated skeletons, and passed to Open Babel for a 10,000 steps of Steepest Descent minimization using the Universal Force Field. Chain A of hAChE (PDB 6O4W) and hBChE (PDB entry 6F7Q) [31] enzyme structures were passed to the Protein Preparation Wizard interface of Maestro, where water molecules were removed from the protein target and hydrogen atoms were added, their positions optimized, and the protonation states of residues determined according to PROPKA prediction at pH 7.0. AMBER UNITED force field electrostatic charges [32] were loaded on the protein structure, while the molcharge complement of QUACPAC [33] was used in order to achieve Marsili–Gasteiger charges for the inhibitors. Affinity maps were first calculated on a 0.375 Å spaced 85 × 85 × 85 Å^3^ cubic box, centered on the inhibitors’ crystallized structures, and the accessibility of the binding site was exploited throughout 250 runs of the Lamarckian Genetic Algorithm (LGA) implemented in AUTODOCK 4.2.6 [34]. 

Bridging water molecules were taken into account by using the automated hydration procedure in AUTODOCK [35], and the population size and the number of energy evaluations figures were set to 300 and 10,000,000, respectively. Among all the plausible binding poses, either the best AUTODOCK free energy scoring function pose and/or the most populated cluster was finally selected as representative of the ChEs/inhibitor complex.

### 4.3. Chemistry

Starting materials, reagents, and analytical grade solvents were purchased from Sigma-Aldrich, Milan, Italy. The purity of all the intermediates, checked by HPLC, was always better than 95%. All the newly prepared and tested compounds showed purity higher than 95% (elemental analysis). Elemental analyses were performed on the EuroEA 3000 analyzer for the final compounds tested as MAOs and ChEs inhibitors. The measured values for C, H, and N agreed to within ±0.40% of the theoretical values. Column chromatography was performed using Merck silica gel 60 (0.063–0.200 mm, 70–230 mesh). Flash chromatographic separations were performed on a Biotage SP1 purification system using flash cartridges prepacked with KP-Sil 32–63 μm, 60 Å silica. All reactions were routinely checked by TLC using Merck Kieselgel 60 F_254_ aluminum plates and visualized by UV light or iodine. Regarding the reaction requiring the use of dry solvents, the glassware was flame-dried and then cooled under a stream of dry argon before the use. Nuclear magnetic resonance spectra were recorded on a Varian Mercury 300 instrument (at 300 MHz) or on an Agilent Technologies 500 apparatus (at 500 MHz) at ambient temperature in the specified deuterated solvent. Chemical shifts (δ) are quoted in parts per million (ppm) and are referenced to the residual solvent peak. The coupling constants *J* are given in Hertz (Hz). The following abbreviations were used: s (singlet), d (doublet), dd (doublet of doublet), t (triplet), m (multiplet), br (broad signal); signals due to OH and NH protons were located by deuterium exchange with D_2_O. The attribution of ^1^H NMR signals was supported by comparison with literature data of close analogues. Melting points for solid final compounds were determined by the capillary method on a Stuart Scientific SMP3 electrothermal apparatus and are uncorrected. HRMS experiments were performed with a dual electrospray interface (ESI) and a quadrupole time-of-flight mass spectrometer (Q-TOF, Agilent 6530 Series Accurate-Mass Quadrupole Time-of-Flight LC/MS, Agilent Technologies Italia S.p.A., Cernusco sul Naviglio, Italy). Full-scan mass spectra were recorded in the mass/charge (*m*/*z*) range 50–3000 Da. The following derivatives (intermediates or tested final compounds) have been already described in the literature: 4-(chloromethyl)-7-hydroxy-2*H*-chromen-2-one (**1a**) [13], 4-(chloromethyl)-7-hydroxy-8-methyl-2*H*-chromen-2-one (**1d**) [36], 7-[(3-chlorobenzyl)oxy]-4-(chloromethyl)-2*H*-chromen-2-one (**2a**) [36], 4-(chloromethyl)-7-[(3-fluorobenzyl)oxy]-2*H*-chromen-2-one (**2b**) [10], 7-[(3-bromobenzyl)oxy]-4-(chloromethyl)-2*H*-chromen-2-one (**2c**) [21], 7-(benzyloxy)-4-(chloromethyl)-2*H*-chromen-2-one (**2d**) [10], 7-(1,3-benzodioxol-5-ylmethoxy)-4-(chloromethyl)-2*H*-chromen-2-one (**2g**) [10], 7-[(3-chlorobenzyl)oxy]-4-[(methylamino)methyl]-2*H*-chromen-2-one (**3**) [10], 7-(1,3-benzodioxol-5-ylmethoxy)-4-[(methylamino)methyl]-2*H*-chromen-2-one (**6**) [10], 7-(benzyloxy)-4-[(methylamino)methyl]-2*H*-chromen-2-one (**7**) [10], 7-[(3-bromobenzyl)oxy]-4-[(methylamino)methyl]-2*H*-chromen-2-one (**8**) [21], 7-[(3-fluorobenzyl)oxy]-4-[(methylamino)methyl]-2*H*-chromen-2-one (**9**) [10], 4-[(benzylamino)methyl]-7-[(3-chlorobenzyl)oxy]-2*H*-chromen-2-one (**13**) [10], 4-{[benzyl(methyl)amino]methyl}-7-[(3-chlorobenzyl)oxy]-2*H*-chromen-2-one (**14**) [10], 7-[(3-chlorobenzyl)oxy]-4-(pyrrolidin-1-ylmethyl)-2*H*-chromen-2-one (**15**) [21], 7-[(3-chlorobenzyl)oxy]-4-(morpholin-4-ylmethyl)-2*H*-chromen-2-one (**17**) [21], 4-(aminomethyl)-7-[(3-chlorobenzyl)oxy]-2*H*-chromen-2-one (**18**) [10], 7-[(3-chlorobenzyl)oxy]-4-[(dimethylamino)methyl]-2*H*-chromen-2-one (**19**) [10], 7-[(3-chlorobenzyl)oxy]-4-[(propylamino)methyl]-2*H*-chromen-2-one (**20**) [21], 4-[(butylamino)methyl]-7-[(3-chlorobenzyl)oxy]-2*H*-chromen-2-one (**21**) [21].

General procedure for the preparation of starting 7-hydroxy-4-chloromethyl-2*H*-chromen-2-ones **1b**–**c**: Prepared according to procedure described in ref. [10] or [36]. The appropriate resorcinol (55 mmol) was dissolved in conc. sulfuric acid (60 mL) at 0 °C. Then ethyl 4-chloroacetoacetate (8.2 mL, 50 mmol) was added dropwise while cooling. After stirring for 1 h at 0 °C (for **1c**) or for 24 h at room temperature (for **1b**), the reaction mixture was poured onto ice. The precipitate was collected, filtered, washed with abundant water, and purified as detailed below, when necessary.

*4-(chloromethyl)-7-hydroxy-6-methyl-2H-chromen-2-one* (**1b**): Prepared from 2,4-dihydroxytoluene (6.8 g, 55 mmol). Used without further purification. Brown solid; yield: 8.2 g, 73%. ^1^H NMR (300 MHz, Acetone-*d_6_*) δ: 2.27 (s, 3H, CH_3_), 4.91 (s, 2H, CH_2_Cl), 6.38 (s, 1H, H-3_coum_), 6.75 (s, 1H, H-8_coum_), 7.61 (s, 1H, H-5_coum_), OH not detectable.

*6-Chloro-4-(chloromethyl)-7-hydroxy-2H-chromen-2-one* (**1c**): Prepared from 4-chlororesorcinol (8.0 g, 55 mmol). Purified through flash chromatography (elution gradient: methanol in chloroform 0%→5%). White solid; yield: 5.8 g, 47%. ^1^H NMR (300 MHz, DMSO-d_6_) δ: 4.97 (s, 2H, CH_2_Cl), 6.47 (s, 1H, H-3_coum_), 6.92 (s, 1H, H-8_coum_), 7.84 (s, 1H, H-5_coum_), 11.48 (s, 1H, dis. with D_2_O, OH).

*[3-(Dimethylamino)phenyl]methanol* (**1e**): 3-(Dimethylamino)benzoic acid (3.3 g, 20 mmol) was dissolved in dry THF (100 mL) and then 1.0 M BH_3_ solution in THF (60 mL, 60 mmol) was added dropwise while cooling at 0 °C. The reaction mixture was slowly warmed at ambient temperature and kept under magnetic stirring for 24 h. After cooling through an external ice bath, the excess borane was quenched by slowly adding methanol. The solvents were removed under reduced pressure. The resulting mixture was partitioned with ethyl acetate (200 mL) and 2 N aq. NaOH (3 × 50 mL), and washed with brine (1 × 50 mL). The organic layer was dried over Na_2_SO_4_, concentrated under vacuum and purified through flash chromatography (elution gradient: methanol in chloroform 0% → 5%). Yellow oil; yield: 2.7 g, 89%. ^1^H NMR (300 MHz, CDCl_3_) δ: 1.67 (br, 1H, dis. with D_2_O, OH), 2.96 (s, 6H, N(CH_3_)_2_), 4.65 (s, 2H, *CH_2_*OH), 6.66–6.75 (m, 3H, Ar), 7.21–7.23 (m, 1H, Ar).

*4-(Chloromethyl)-7-{[3-(dimethylamino)benzyl]oxy}-2H-chromen-2-one* (**2e**): Phenol **1a** (1.6 g, 7.5 mmol) was dissolved in dry THF (60 mL) followed by the addition of **1e** (2.3 g, 15 mmol), DIAD (3.0 g, 15 mmol) and a solution of PPh_3_ (3.9 g, 15 mmol) in THF (15 mL), via syringe at 0 °C. The mixture was stirred at room temperature overnight and then concentrated to dryness under rotary evaporation. The resulting oil residue was purified through flash chromatography (elution gradient: EtOAc in *n*-hexane 0% → 80%). Brown solid; yield: 0.80 g, 31%. ^1^H NMR (300 MHz, CDCl_3_) δ: 2.97 (s, 6H, N(CH_3_)_2_), 4.61 (s, 2H, CH_2_Cl), 5.10 (s, 2H, CH_2_O), 6.39 (s, 1H, H-3_coum_), 6.71–6.76 (m, 3H, Ar), 6.93 (d, *J* = 2.5 Hz, 1H, H-8_coum_), 6.97 (dd, *J* = 8.8, 2.5 Hz, 1H, H-6_coum_), 7.20–7.23 (m, 1H, Ar), 7.56 (d, *J* = 8.8 Hz, 1H, H-5_coum_).

General procedure for the preparation of 7-benzyloxy-4-chloromethyl-2*H*-chromen-2-ones **2f**, **2h**–**j**: The appropriate 7-OH-coumarin **1a**–**d** (20 mmol) was suspended in ethanol (100 mL), before adding DIEA (7.0 mL, 40 mmol) and the suitable benzyl bromide (40 mmol). The mixture was refluxed until reaction completion (TLC control, 1.5–5 h) and the solvent was removed under rotary evaporation. The resulting crude was purified through flash chromatography as indicated below.

*4-(Chloromethyl)-7-[(3,5-dimethoxybenzyl)oxy]-2H-chromen-2-one* (**2f**): Prepared from **1a** (4.2 g, 20 mmol) and 3,5-dimethoxybenzyl bromide (9.2 g, 40 mmol). Purified through flash chromatography (elution gradient: chloroform in *n*-hexane 60% → 100%). White solid; yield: 4.4 g, 61%. ^1^H NMR (300 MHz, DMSO-*d_6_*) δ: 3.73 (s, 6H, OCH_3_), 4.97 (s, 2H, CH_2_Cl), 5.16 (s, 2H, CH_2_O), 6.43–6.45 (m, 1H, Ar), 6.53 (s, 1H, H-3_coum_), 6.59-6.63 (m, 2H, Ar), 7.03–7.12 (m, 2H, H-6_coum_ + H-8_coum_), 7.75 (d, J = 8.4 Hz, 1H, H-5_coum_).

*7-[(3-Chlorobenzyl)oxy]-4-(chloromethyl)-6-methyl-2H-chromen-2-one* (**2h**): Prepared from **1b** (4.5 g, 20 mmol) and 3-chlorobenzyl bromide (5.3 mL, 40 mmol). Purified through flash chromatography (elution gradient: EtOAc in *n*-hexane 10% → 60%). Yellow solid; yield: 4.0 g, 57%. ^1^H NMR (300 MHz, DMSO-*d_6_*) δ: 2.12 (s, 3H, CH_3_), 4.96 (s, 2H, CH_2_Cl), 5.26 (s, 2H, CH_2_O), 6.47 (s, 1H, H-3_coum_), 7.11 (s, 1H, H-8_coum_), 7.39–7.46 (m, 3H, Ar), 7.53 (s, 1H, Ar), 7.65 (s, 1H, H-5_coum_).

*6-Chloro-7-[(3-chlorobenzyl)oxy]-4-(chloromethyl)-2H-chromen-2-one* (**2i**): Prepared from **1c** (4.9 g, 20 mmol) and 3-chlorobenzyl bromide (5.3 mL, 40 mmol). Purified through flash chromatography (elution gradient: chloroform in *n*-hexane 50% → 80%). White solid; yield: 6.6 g, 89%. ^1^H NMR (300 MHz, DMSO-*d_6_*) δ: 5.01 (s, 2H, CH_2_Cl), 5.35 (s, 2H, CH_2_O), 6.54 (s, 1H, H-3_coum_), 7.36 (s, 1H, H-8_coum_), 7.38–7.45 (m, 3H, Ar), 7.55 (s, 1H, Ar), 7.95 (s, 1H, H-5_coum_).

*7-[(3-Chlorobenzyl)oxy]-4-(chloromethyl)-8-methyl-2H-chromen-2-one* (**2j**): Prepared from **1d** (4.5 g, 20 mmol) and 3-chlorobenzyl bromide (5.3 mL, 40 mmol). Purified through flash chromatography (elution gradient: chloroform in *n*-hexane 10% → 80%). Off-white solid; yield: 5.7 g, 81%. ^1^H NMR (300 MHz, Acetone-*d_6_*) δ: 2.24 (s, 3H, CH_3_), 4.98 (s, 2H, CH_2_Cl), 5.30 (s, 2H, CH_2_O), 6.50 (s, 1H, H-3_coum_), 7.15 (d, *J* = 8.8 Hz, 1H, H-6_coum_), 7.41–7.46 (m, 3H, Ar), 7.54 (s, 1H, Ar), 7.67 (*d*, *J* = 8.8 Hz, 1H, H-5_coum_).

General procedure for the preparation of final compounds **4**–**5**, **10**–**12**. To a stirred 2.0 N methylamine solution in THF (10 mL), the suitable chloride **2e**–**f**, **2h**–**j** was added portionwise (1.0 mmol, six aliquots, 1 h time interval) under an Ar atmosphere. The mixture was stirred at room temperature for 6–24 h until disappearance of the starting material (TLC control). After the evaporation of excess amine and THF under reduced pressure, the resulting crude was purified as indicated below.

*7-{[3-(Dimethylamino)benzyl]oxy}-4-[(methylamino)methyl]-2H-chromen-2-one* (**4**): Prepared from **2e** (0.34 g, 1.0 mmol). Purified through flash chromatography (eluent: EtOAc). Yellow solid; low-melting; yield: 0.27 g, 80%. ^1^H-NMR (300 MHz, DMSO-*d_6_*) δ: 2.33 (s, 3H, CH_2_NH*CH_3_*), 2.86 (s, 6H, N(CH_3_)_2_), 3.80 (s, 2H, *CH_2_*NHCH_3_), 5.17 (s, 2H, CH_2_O), 6.29 (s, 1H, H-3_coum_), 6.66–6.75 (m, 3H, Ar), 7.00 (dd, *J* = 8.8, 2.5 Hz, 1H, H-6_coum_), 7.04 (d, *J* = 2.5 Hz, 1H, H-8_coum_), 7.21–7.23 (m, 1H, Ar), 7.73 (d, *J* = 8.8 Hz, 1H, H-5_coum_), NH not detected. Anal. (C_20_H_22_N_2_O_3_) calcd. % C, 70.99; H, 6.55; N, 8.28. Found % C, 71.12; H, 6.46; N, 8.18. HRMS (Q-TOF) calcd. for C_20_H_22_N_2_O_3_ [*M* + Na]^+^ *m*/*z* 361.1523, found 361.1524.

*7-[(3,5-Dimethoxybenzyl)oxy]-4-[(methylamino)methyl]-2H-chromen-2-one* (**5**): Prepared from **2f** (0.36 g, 1.0 mmol). Purified through flash chromatography (eluent: EtOAc). Yield: 0.22 g, 61%. ^1^H-NMR (300 MHz, DMSO-*d*_6_) δ: 2.32 (s, 3H, CH_2_NH*CH_3_*), 3.72 (s, 6H, OCH_3_), 3.81 (s, 2H, *CH_2_*NHCH_3_), 5.14 (s, 2H, CH_2_O), 6.28 (s, 1H, H-3_coum_), 6.44 (t, *J* = 2.2 Hz, 1H, Ar), 6.60 (d, *J* = 2.2 Hz, 2H, Ar), 6.99 (dd, *J* = 8.8 Hz, 2.5 Hz, 1H, H-6_coum_), 7.03 (d, *J* = 2.5 Hz, 1H, H-8_coum_), 7.73 (d, *J* = 8.8 Hz, 1H, H-5_coum_), NH not detected. Anal. (C_20_H_21_NO_5_) calcd. % C, 67.59; H, 5.96; N, 3.94. Found % C, 67.68; H, 6.01; N, 3.87. HRMS (Q-TOF) calcd. for C_20_H_21_NO_5_ [*M* + Na]^+^ *m*/*z* 378.1312, found 378.1312.

*7-[(3-Chlorobenzyl)oxy]-8-methyl-4-[(methylamino)methyl]-2H-chromen-2-one* (**10**): Prepared from **2j** (0.35 g, 1.0 mmol). Purified through flash chromatography (eluent: EtOAc). White solid; mp: 145–146 °C; yield: 0.11 g, 33%. ^1^H-NMR (300 MHz, DMSO-*d*_6_) δ: 2.23 (s, 3H, CH_3_), 2.32 (s, 3H, CH_2_NH*CH_3_*), 3.81 (s, 2H, *CH_2_*NHCH_3_), 5.27 (s, 2H, CH_2_O), 6.29 (s, 1H, H-3_coum_), 7.06 (d, *J* = 8.8 Hz, 1H, H-6_coum_), 7.39–7.44 (m, 3H, Ar), 7.52 (s, 1H, Ar), 7.62 (d, *J* = 8.8 Hz, 1H, H-5_coum_), NH not detected. Anal. (C_19_H_18_ClNO_3_) calcd. % C, 66.38; H, 5.28; N, 4.07. Found % C, 66.45; H, 5.40; N, 4.02. HRMS (Q-TOF) calcd. for C_19_H_18_ClNO_3_ [*M* + Na]^+^ *m*/*z* 366.0867, found 366.0869.

*7-[(3-Chlorobenzyl)oxy]-6-methyl-4-[(methylamino)methyl]-2H-chromen-2-one hydrochloride* (**11**): Prepared from **2h** (0.35 g, 1.0 mmol). Purified through flash chromatography (eluent: EtOAc) and transformed into the corresponding hydrochloride salt by treatment with 4.0 N HCl solution in 1,4-dioxane. White solid; mp > 230 °C; yield: 0.17 g, 44%. ^1^H-NMR (300 MHz, DMSO-*d*_6_) δ: 2.27 (s, 3H, CH_3_), 2.68 (s, 3H, CH_2_NH_2_^+^*CH_3_*), 4.42 (s, 2H, *CH_2_*NH_2_^+^CH_3_), 5.28 (s, 2H, CH_2_O), 6.42 (s, 1H, H-3_coum_), 7.14 (s, 1H, H-8_coum_), 7.38–7.45 (m, 3H, Ar), 7.53 (s, 1H, Ar), 7.66 (s, 1H, H-5_coum_), 9.88 (br s, 2H, dis. with D_2_O, NH_2_^+^). Anal. (C_19_H_19_Cl_2_NO_3_) calcd. % C, 60.01; H, 5.04; N, 3.68. Found % C, 60.12; H, 5.03; N, 3.72. HRMS (Q-TOF) calcd. for C_19_H_18_ClNO_3_ [*M* + Na]^+^ *m*/*z* 366.0867, found 366.0870.

*6-Chloro-7-[(3-chlorobenzyl)oxy]-4-[(methylamino)methyl]-2H-chromen-2-one* (**12**): Prepared from **2i** (0.37 g, 1.0 mmol). Purified through flash chromatography (eluent: EtOAc). White solid; mp: 176–177 °C; yield: 0.13 g, 37%. ^1^H-NMR (300 MHz, DMSO-*d*_6_) δ: 2.31 (s, 3H, NH*CH_3_*), 3.81 (s, 2H, *CH_2_*NH), 5.33 (s, 2H, CH_2_O), 6.34 (s, 1H, H-3_coum_), 7.31 (s, 1H, H-8_coum_), 7.42–7.48 (m, 3H, Ar), 7.54 (s, 1H, Ar), 7.93 (s, 1H, H-5_coum_), NH not detected. Anal. (C_18_H_15_Cl_2_NO_3_) calcd. % C, 59.36; H, 4.15; N, 3.85. Found % C, 59.48; H, 4.20; N, 3.83. HRMS (Q-TOF) calcd. for C_18_H_15_Cl_2_NO_3_ [*M* + Na]^+^ *m*/*z* 386.0321, found 386.0324.

General procedure for the preparation of final compounds **16**, **22**–**27**. After dissolving chloride **2a** or **2f** (1.0 mmol) in 8 mL of dry THF (dry acetone or DMF for the synthesis of **16** and **24**, respectively), an excess amine was added, followed by a proton scavenger (K_2_CO_3_ for **16**, **22** and **23**: 0.28 g, 2.0 mmol; DIEA for **24**–**27**: 1.4 mL, 8.0 mmol). The reaction mixtures were kept under magnetic stirring at room temperature for 24–72 h (for **16**, **22**, **23**) or heated at 80 °C for 4–6 h (**24**–**27**). After evaporation of the solvent under rotary evaporation, the resulting crude was purified as indicated below.

*7-[(3-Chlorobenzyl)oxy]-4-[(4-methylpiperazin-1-yl)methyl]-2H-chromen-2-one* (**16**): Prepared from **2a** (0.34 g, 1.0 mmol) and 1-methylpiperazine (0.17 mL, 1.5 mmol). The crude was partitioned between brine (80 mL) and diethyl ether (50 mL). The organic phase was dried over Na_2_SO_4_ and concentrated to dryness. Crystallization from hot ethanol afforded the title compound **16**. Pale yellow solid; mp: 133–134 °C; yield: 0.19 g, 47%. ^1^H-NMR (300 MHz, DMSO-*d*_6_) δ: 2.13 (s, 3H, NCH_3_), 2.23–2.36 (m, 4H, N-cycle), 2.40–2.46 (m, 4H, N-cycle), 3.61 (s, 2H, CH_2_N), 5.23 (s, 2H, CH_2_O), 6.28 (s, 1H, H-3_coum_), 7.01 (dd, *J* = 8.8, 2.5 Hz, 1H, H-6_coum_), 7.06 (d, *J* = 2.5 Hz, 1H, H-8_coum_), 7.39–7.43 (m, 3H, Ar), 7.53 (s, 1H, Ar), 7.84 (d, *J* = 8.8 Hz, 1H, H-5_coum_). Anal. (C_22_H_23_ClN_2_O_3_) calcd. % C, 66.24; H, 5.81; N, 7.02. Found % C, 66.41; H, 5.90; N, 6.93. HRMS (Q-TOF) calcd. for C_22_H_23_ClN_2_O_3_ [*M* + Na]^+^ *m*/*z* 421.1289, found 421.1289.

*7-[(3-Chlorobenzyl)oxy]-4-[(prop-2-yn-1-ylamino)methyl]-2H-chromen-2-one* (**22**): Prepared from **2a** (0.34 g, 1.0 mmol) and propargylamine (0.64 mL, 10 mmol). Purified through flash chromatography (elution gradient: EtOAc in *n*-hexane 20% → 100%). White solid; mp: 114–115 °C; yield: 0.24 g, 69%. ^1^H-NMR (300 MHz, DMSO-*d*_6_) δ: 2.71–2.76 (m, 1H, C≡CH), 3.37 (s, 2H, NH*CH_2_*C≡CH), 3.90 (s, 2H, CH_2_NH*CH_2_*C≡CH), 5.23 (s, 2H, CH_2_O), 6.28 (s, 1H, H-3_coum_), 7.01 (dd, *J* = 8.8, 2.5 Hz, 1H, H-6_coum_), 7.07 (d, *J* = 2.5 Hz, 1H, H-8_coum_), 7.37–7.43 (m, 3H, Ar), 7.53 (s, 1H, Ar), 7.74 (d, J = 8.8 Hz, 1H, H-5_coum_), NH not detected. Anal. (C_20_H_16_ClNO_3_) calcd. % C, 67.90; H, 4.56; N, 3.96. Found % C, 68.02; H, 4.50; N, 3.99. HRMS (Q-TOF) calcd. for C_20_H_16_ClNO_3_ [*M* + Na]^+^ *m*/*z* 376.0711, found 376.0713.

*7-[(3-Chlorobenzyl)oxy]-4-{[methyl(prop-2-yn-1-yl)amino]methyl}-2H-chromen-2-one hydrochloride* (**23**): Prepared from **2a** (0.34 g, 1.0 mmol) and *N*-methylpropargylamine (0.25 mL, 3.0 mmol). Purified through flash chromatography (elution gradient: EtOAc in n-hexane 10% → 70%) and transformed into the corresponding hydrochloride salt by treatment with 4.0 N HCl solution in 1,4-dioxane. White solid; mp: 183–184 °C (dec.); yield: 0.36 g, 88%. ^1^H-NMR (300 MHz, D_2_O+DMSO-*d*_6_) δ: 2.65 (s, 1H, C≡CH), 2.70 (s, 3H, NH^+^(*CH_3_*)), 3.95 (s, 2H, NH^+^(CH_3_)*CH_2_*C≡CH), 4.22 (s, 2H, *CH_2_*NH^+^(CH_3_)CH_2_C≡CH), 5.25 (s, 2H, CH_2_O), 6.53 (s, 1H, H-3_coum_), 7.05 (dd, *J* = 8.8, 2.2 Hz, 1H, H-6_coum_), 7.12 (d, *J* = 2.2 Hz, 1H, H-8_coum_), 7.37–7.43 (m, 3H, Ar), 7.53 (s, 1H, Ar), 7.88 (d, *J* = 8.8 Hz, 1H, H-5_coum_). Anal. (C_21_H_19_Cl_2_NO_3_) calcd. % C, 62.39; H, 4.74; N, 3.46. Found % C, 62.51; H, 4.70; N, 3.31. HRMS (Q-TOF) calcd. for C_21_H_18_ClNO_3_ [*M* + Na]^+^ *m*/*z* 390.0867, found 390.0868.

*N^2^-({7-[(3-chlorobenzyl)oxy]-2-oxo-2H-chromen-4-yl}methyl)glycinamide* (**24**): Prepared from **2a** (0.34 g, 1.0 mmol) and glycinamide hydrochloride (0.55 g, 5.0 mmol). Purified through column chromatography (eluent: 5% methanol in chloroform). Off-white solid; mp: 183–184 °C; yield: 0.24 g, 64%. ^1^H-NMR (300 MHz, DMSO-*d*_6_) δ: 3.12 (s, 2H, CH_2_CO), 3.87 (s, 2H, *CH_2_*NH), 5.22 (s, 2H, CH_2_O), 6.35 (s, 1H, H-3_coum_), 7.00 (dd, *J* = 8.8, 2.5 Hz, 1H, H-6_coum_), 7.07 (m, 2H, H-8_coum_ + CONH_a_), 7.31 (s, 1H, CONH_b_), 7.38–7.43 (m, 3H, Ar), 7.53 (s, 1H, Ar), 7.73 (d, *J* = 8.8 Hz, 1H, H-5_coum_), aminic NH not detected. Anal. (C_19_H_17_ClN_2_O_4_) calcd. % C, 61.21; H, 4.60; N, 7.51. Found % C, 61.33; H, 4.64; N, 7.45. HRMS (Q-TOF) calcd. for C_19_H_17_ClN_2_O_4_ [*M* + Na]^+^ *m*/*z* 395.0769, found 395.0769.

*N^2^-({7-[(3-Chlorobenzyl)oxy]-2-oxo-2H-chromen-4-yl}methyl)-N^1^-methylglycinamide* (**25**): Prepared from **2a** (0.34 g, 1.0 mmol) and 2-amino-*N*-methylacetamide hydrochloride (0.62 g, 5.0 mmol). Purified through column chromatography (eluent: 10% methanol in chloroform). White solid; mp: 168–169 °C; yield: 0.24 g, 61%. ^1^H-NMR (300 MHz, DMSO-*d*_6_) δ: 2.58 (d, *J* = 4.7 Hz, 3H, CONH*CH_3_*), 3.14 (s, 2H, *CH_2_*CO), 3.86 (s, 2H, CH_2_NH), 5.22 (s, 2H, CH_2_O), 6.32 (s, 1H, H-3_coum_), 7.00 (dd, *J* = 8.8, 2.5 Hz, 1H, H-6_coum_), 7.07 (d, *J* = 2.5 Hz, 1H, H-8_coum_), 7.38-7.43 (m, 3H, Ar), 7.52 (s, 1H, Ar), 7.72 (d, *J* = 8.8 Hz, 1H, H-5_coum_), 7.73–7.78 (m, 1H, CONH), aminic NH not detected. Anal. (C_20_H_19_ClN_2_O_4_) calcd. % C, 62.10; H, 4.95; N, 7.24. Found % C, 62.42; H, 4.79; N, 7.15. HRMS (Q-TOF) calcd. for C_20_H_19_ClN_2_O_4_ [*M* + Na]^+^ *m*/*z* 409.0926, found 409.0942.

*N^2^-({7-[(3-Chlorobenzyl)oxy]-2-oxo-2H-chromen-4-yl}methyl)-*N*^1^,*N*^1^-dimethylglycinamide* (**26**): Prepared from **2a** (0.34 g, 1.0 mmol) and 2-amino-N,N-dimethylacetamide acetate (0.81 g, 5.0 mmol). Purified through column chromatography (eluent: 5% methanol in chloroform). White solid; mp: 134–135 °C; yield: 0.21 g, 53%. ^1^H-NMR (300 MHz, DMSO-*d*_6_) δ: 2.82 (s, 3H, NCH_3_), 2.87 (s, 3H, NCH_3_), 3.40 (s, 2H, CH_2_CO), 3.84 (s, 2H, *CH_2_*NH), 5.22 (s, 2H, CH_2_O), 6.30 (s, 1H, H-3_coum_), 7.00 (dd, *J* = 8.8, 2.5 Hz, 1H, H-6_coum_), 7.06 (d, *J* = 2.5 Hz, 1H, H-8_coum_), 7.38–7.42 (m, 3H, Ar), 7.52 (s, 1H, Ar), 7.75 (d, *J* = 8.8 Hz, 1H, H-5_coum_), aminic NH not detected. Anal. (C_21_H_21_ClN_2_O_4_) calcd. % C, 62.92; H, 5.28; N, 6.99. Found % C, 63.09; H, 5.20; N, 7.06. HRMS (Q-TOF) calcd. for C_21_H_21_ClN_2_O_4_ [*M* + Na]^+^ *m*/*z* 423.1082, found 423.1089.

*N^2^-({7-[(3,5-dimethoxybenzyl)oxy]-2-oxo-2H-chromen-4-yl}methyl)glycinamide* (**27**): Prepared from **2f** (0.36 g, 1.0 mmol) and glycinamide hydrochloride (0.55 g, 5.0 mmol). Purified through column chromatography (eluent: 10% methanol in chloroform). White solid; mp: 130–131 °C; yield: 0.29 g, 74%. ^1^H-NMR (500 MHz, DMSO-*d*_6_) δ: 3.32 (s, 2H, CH_2_CO), 3.73 (s, 6H, OCH_3_), 3.88 (s, 2H, CH_2_NH), 5.15 (s, 2H, CH_2_O), 6.36 (s, 1H, H-3_coum_), 6.45 (t, *J* = 2.4 Hz, 1H, Ar), 6.62 (d, *J* = 2.4 Hz, 2H, Ar), 7.00 (dd, *J* = 8.8, 2.5 Hz, 1H, H-6_coum_), 7.05 (m, 2H, H-8_coum_ + CONH_a_), 7.31 (s, 1H, CONH_b_), 7.73 (d, *J* = 8.8 Hz, 1H, H-5_coum_), aminic NH not detected. Anal. (C_21_H_22_N_2_O_6_) calcd. % C, 63.31; H, 5.57; N, 7.03. Found % C, 63.33; H, 5.51; N, 6.98. HRMS (Q-TOF) calcd. for C_21_H_22_N_2_O_6_ [*M* + Na]^+^ *m*/*z* 421.1370, found 421.1371.

## 5. Conclusions

In the present work, we aimed at finding novel multipotent compounds capable of inhibiting AD-related enzymatic targets, namely MAO B and ChEs, by exploring structural modifications around the 4-aminomethyl-7-benzyloxy-2*H*-chromen-2-one core at the basic protonatable head or at the benzyloxy tail. A larger activity window was observed in the case of MAO B, where structural requirements, basically the influence of steric bulk within the protonatable group, were confirmed.

Unfortunately, the structural modifications explored herein did not succeed in tightening the binding with ChEs and in improving inhibition to a higher extent. The kinetic analysis indicated a mixed-type inhibition for AChE, which suggests the possibility for ligands to bind peripheral subsites. Docking simulations corroborated these results, highlighting productive binding interactions at the PAS region of both ChEs and suggesting the inability of these molecules to deeply lock into the catalytic region of AChE. Irrespective of the basic moiety, the binding contacts were limited to PAS residues and the coumarin derivatives were unable to reach and interact with the catalytic Ser203 and the CAS (e.g., Trp86). 

We succeeded in identifying different coumarins with an interesting multiple activity showing nanomolar activity toward MAO B along with AChE and/or BChE inhibition in the low micromolar range (e.g., **3**, **6**, **8**, **9**, **20**, **22**–**27**). A more balanced multi-targeting compound was identified in derivative **16** (IC_50_ = 0.27 μM, 3.1 μM and 2.3 μM toward MAO B, AChE, and BChE, respectively). In light of these data, further hit optimization programs along with deeper in vitro and computational studies will be fostered to achieve balanced profiles with higher ChEs affinity and to rule out off-target activities (e.g., through off-target battery docking).

## Figures and Tables

**Table 1:** Biological data of coumarin derivatives **3**–**27**.

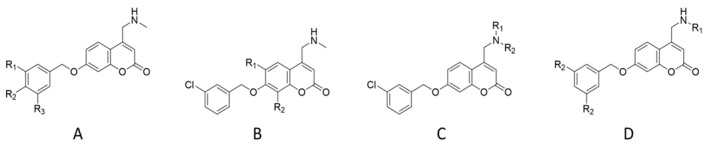								
**General structure**	**entry**	**R_1_**	**R_2_**	**R_3_**	**MAO A ^a,b^**	**MAO B ^a,b^**	**AChE ^a,c^**	**BChE ^a,d^**
A	**3**	Cl	H	H	0.65 ± 0.02	0.0039 ± 0.0008	6.2 ± 0.7	43 ± 5%
	**4**	NMe_2_	H	H	49 ± 1%	0.57 ± 0.05	6.0 ± 1.5	34 ± 5%
	**5**	OMe	H	OMe	1.0 ± 0.1	0.067 ± 0.005	11 ± 2	20 ± 4%
	**6**	–OCH_2_O–		H	1.2 ± 0.1	0.0075 ± 0.0001	8.5 ± 1.8	3.4 ± 2.1
	**7**	H	H	H	0.53 ± 0.01	0.0021 ± 0.0002	26 ± 5%	17 ± 1%
	**8**	Br	H	H	2.31 ± 0.04	0.0022 ± 0.0001	2.00 ± 0.04	2.9 ± 0.4
	**9**	F	H	H	1.74 ± 0.04	0.0013 ± 0.0001	2.7 ± 1.0	4.8 ± 0.3
**General structure**	**entry**	**R_1_**		**R_2_**	**MAO A**	**MAO B**	**AChE**	**BChE**
B	**10**	H		Me	1.0 ± 0.1	0.17 ± 0.01	7.2 ± 0.6	46 ± 2%
	**11**	Me		H	0.58 ± 0.03	0.068 ± 0.002	47.1 ± 0.3%	48.1 ± 0.6%
	**12**	Cl		H	48 ± 1%	1.0 ± 0.1	7.9 ± 0.4	27 ± 2%
**General structure**	**entry**	**R_1_**		**R_2_**	**MAO A**	**MAO B**	**AChE**	**BChE**
C	**13**	Bn		H	24 ± 3%	0.103 ± 0.001	17 ± 1	3.4 ± 1.3
	**14**	Bn		Me	28 ± 1%	0.332 ± 0.026	16 ± 1	6.3 ± 0.3
	**15**	pyrrolidin-1-yl			1.12 ± 0.01	0.152 ± 0.020	6.6 ± 0.9	14 ± 2%
	**16**	4-methylpiperazin-1-yl			32 ± 5%	0.273 ± 0.011	3.1 ± 0.6	2.3 ± 0.9
	**17**	morpholin-1-yl			32 ± 3%	0.172 ± 0.001	6.6 ± 1.1	46 ± 2%
	**18**	H		H	46 ± 3%	0.066 ± 0.015	17 ± 4	20 ± 7%
	**19**	Me		Me	9.2 ± 0.7	0.213 ± 0.002	8.3 ± 2.0	7.0 ± 1.1
	**20**	*n*Pr		H	48 ± 1%	0.0094 ± 0.0035	4.9 ± 1.4	3.6 ± 0.8
	**21**	*n*Bu		H	41 ± 3%	0.164 ± 0.002	39 ± 1%	4.8 ± 0.3
	**22**	CH_2_C≡CH		H	1.1 ± 0.1	0.0041 ± 0.0011	2.1 ± 0.2	33 ± 6%
	**23**	CH_2_C≡CH		Me	1.7 ± 0.1	0.084 ± 0.002	5.6 ± 0.7	28 ± 1%
	**24**	CH_2_CONH_2_		H	0.077 ± 0.014	0.061 ± 0.013	2.2 ± 0.7	13 ± 2%
	**25**	CH_2_CONHMe		H	2.6 ± 0.1	0.0011 ± 0.0003	5.3 ± 1.4	<5%
	**26**	CH_2_CONMe_2_		H	2.5 ± 0.5	0.030 ± 0.001	4.3 ± 0.6	34 ± 2%
D	**27**	CH_2_CONH_2_		OMe	5.9 ± 0.5	0.013 ± 0.001	4.4 ± 0.8	41 ± 2%
safinamide					18 ± 3%	0.031 ± 0.001		
donepezil							0.021 ± 0.002	2.3 ± 0.2

^a^ IC_50_ (μM) or % inhibition at 10 μM. Values are the mean ±SEM of three independent experiments. ^b^ Human recombinant monoamine oxidase (MAO). ^c^ acetylcholinesterase (AChE) from electric eel. ^d^ butyrylcholinesterase (BChE) from equine serum.

**Table 2:** Computed drug-like properties for the most active ChEs-MAO B inhibitors.

Entry	MW ^a^	TPSA (Å^2^) ^a^	HBA ^a^	HBD ^a^	cLogP ^b^	BBB ^c^	RO5 violation ^a^
**6**	339.34	69.93	6	1	2.89	+	0
**8**	374.23	51.47	4	1	3.80	+	0
**9**	313.32	51.47	5	1	3.08	+	0
**16**	398.88	45.92	5	0	3.53	+	0
**20**	357.83	51.47	4	1	4.69	+	0

^a^ MW: molecular weight; TPSA: topological polar surface area; HBA: number of hydrogen bond acceptors; HBD: number of hydrogen bond donors; RO5: violation of Lipinsky’s rule of 5. Properties computed with SwissADME web tool. ^b^ Estimated with ACD/Labs 9.04. ^c^ BBB penetration predicted with the BOILED-Egg method through the free web-tools available at http://www.swissadme.ch.

## Supplementary Materials

The following are available online, Figure S1: Michaelis-Menten curves toward eeAChE for compounds **8** and **24**, Figure S2: Superimposition of donepezil/**8** binding poses within human AChE, Figure S3: Different rotamers for mid-gorge Tyr337 in hAChE complexes, Table S1: hAChE docking data for compounds **8**, **24**, donepezil.

## Abbreviations

The following abbreviations are used in this manuscript:

ACh	acetylcholine
AChE	acetylcholinesterase
AD	Alzheimer’s disease
EtOAc	ethyl acetate
EU	European Union
BBB	blood-brain barrier
BChE	butyrylcholinesterase
CAS	catalytic anionic subsite
ChE	cholinesterase
DIAD	diisopropyl azodicarboxylate
DIEA	*N*,*N*-diisopropylethylamine
DMF	*N*,*N*-dimethylformamide
DMSO	dimethylsulfoxide
FAD	flavin adenine dinucleotide
FDA	Food and Drug Administration
GABA	γ-amminobutirric acid
HBA	hydrogen bond acceptor
HBD	hydrogen bond donor
HIV	human immunodeficiency virus
HRMS	high-resolution mass
MAO A	monoamine oxidase A
MAO B	monoamine oxidase B
MTDL	multi-target-directed ligand
PAS	peripheral anionic subsite
PD	Parkinson’s disease
PDB	protein data bank
Q-TOF	quadrupole-time of flight
THF	tetrahydrofuran
TPSA	topological polar surface area
