# Supplementary material for: Chasing ChEs-MAO B Multi-Targeting 4-Aminomethyl-7-Benzyloxy-2H-Chromen-2-ones
Source: Molecules. 2019 Dec 9;24(24):4507. doi: 10.3390/molecules24244507 (PMC6943664; doi:10.3390/molecules24244507)
Supplement: Supplementary file 1 [file molecules-24-04507-s001.pdf]

Chasing ChEs-MAO B Multi-targeting 4-Aminomethyl-7-Benzyloxy-2*H*-Chromen-2-ones

Mariagrazia Rullo[a], Marco Catto[a], Antonio Carrieri[a], Modesto de Candia[a], Cosimo Damiano Altomare[a] and Leonardo Pisani[a,*]

[a]*Department of Pharmacy – Drug Sciences, University of Bari "Aldo Moro", via Orabona 4, Bari – 70125, Italy*

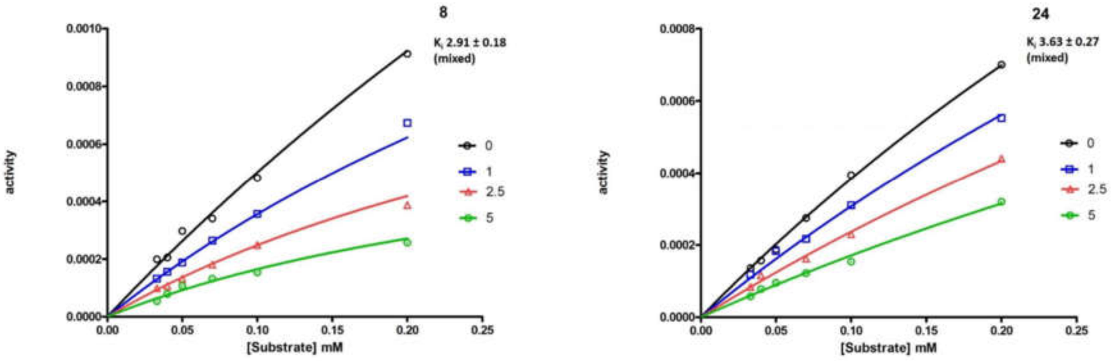

Figure S1. eeAChE enzyme kinetics (Michaelis-Menten curves) for compounds **8** (left) and **24** (right).

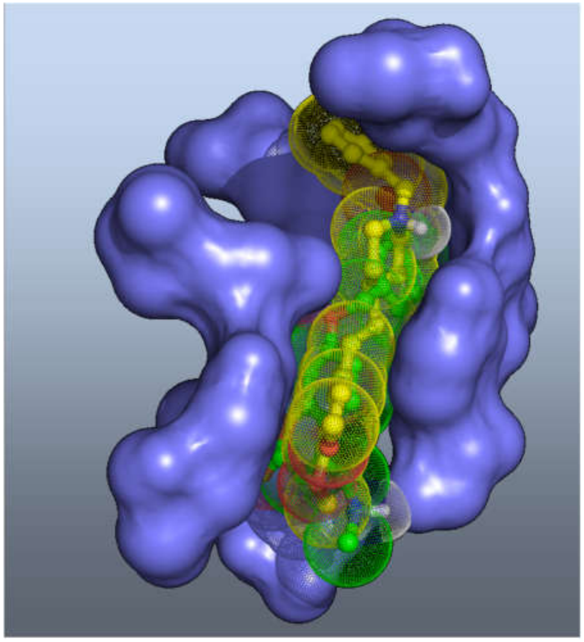

Figure S2. Ball and stick model showing the superimposition of the binding poses of donepezil (yellow) and compound **8** (green) within human AChE (PDB code 6OW4).

* Corresponding author. Tel.: +39-080-544-2803; e-mail: leonardo.pisani@uniba.it

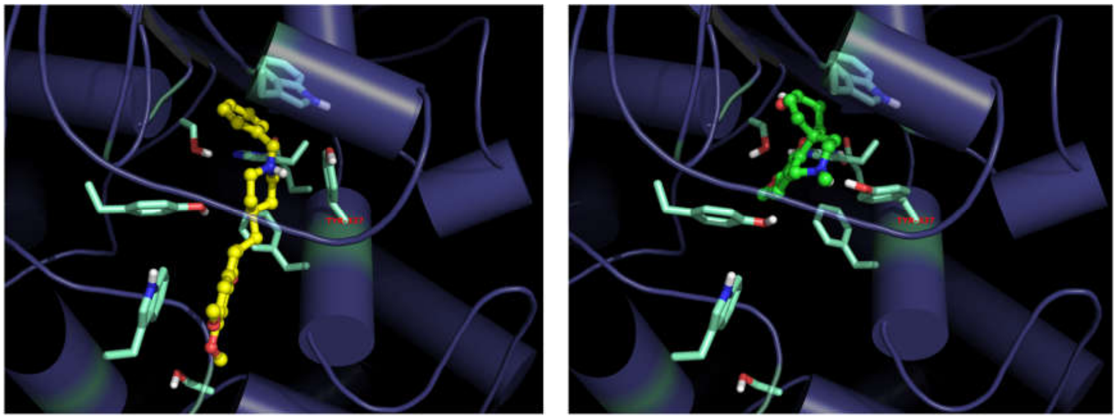

Figure S3. Different rotamers for mid-gorge Tyr337 (cyan) in the crystal structures of hAChE complexed with donepezil (yellow; PDB code 6OW4) and galantamine (green; PDB entry 4EY6) employed in docking simulations. Other key residues, showing no shift between the two models, are shown in cyan.

Table S1. hAChE docking data for compounds **8**, **24** as compared to donepezil.

**entry**	FEB[a]	Population[b]	Efficacy[c]	CSA[d]
**8**	-11.62	59/250	0.505	352.99
**24**	-12.71	26/250	0.489	393.89
**donepezil**	-10.86	55/250	0.388	442.44

[a] Free energy of binding (kcal/mol) as estimated by the AutoDock scoring function.
[b] Frequency of the selected cluster.
[c] Ligand efficacy calculated measured as the absolute FEB value divided by the number of heavy atoms.
[d] Contact surface area in Å^2.
